# Triatominae: does the shape change of non-viable eggs compromise species recognition?

**DOI:** 10.1186/s13071-018-3104-1

**Published:** 2018-10-10

**Authors:** Soledad Santillán-Guayasamín, Anita G. Villacís, Mario J. Grijalva, Jean-Pierre Dujardin

**Affiliations:** 1 Center for Research on Health in Latin America (CISeAL), School of Biological Sciences, Pontifical Catholic University of Ecuador, Calle Pambahacienda s/n y San Pedro del Valle, Campus Nayón, Quito, Ecuador; 2 Infectious and Tropical Disease Institute, Department of Biomedical Sciences, Heritage College of Osteopathic Medicine, Ohio University, Athens, OH 45701 USA; 3 IRD, UMR 177 IRD-CIRAD INTERTRYP, Campus international de Baillarguet, Montpellier, France

**Keywords:** Ecuador, Egg contour, Egg viability, Operculum landmarks, Triatominae

## Abstract

**Background:**

Eggs have epidemiological and taxonomic importance in the subfamily Triatominae, which contains Chagas disease vectors. The metric properties (size and shape) of eggs are useful for distinguishing between close species, or different geographical populations of the same species.

**Methods:**

We examined the effects of egg viability on its metric properties, and the possible consequences on species recognition. Four species were considered: *Panstrongylus chinai*, *P. howardi* and *Triatoma carrioni* (tribe Triatomini), and *Rhodnius ecuadoriensis* (tribe Rhodniini). Digitization was performed on pictures taken when the viability of the egg could not clearly be predicted by visual inspection. We then followed development to separate viable from non-viable eggs, and the metric changes associated with viability status of the eggs were tested for species discrimination (interspecific difference).

**Results:**

The shape of the complete contour of the egg provided satisfactory species classification (95% of correct assignments, on average), with improved scores (98%) when discarding non-viable eggs from the comparisons. Using only non-viable eggs, the scores dropped to 90%. The morphometric differences between viable and non-viable eggs were also explored (intraspecific comparison). A constant metric change observed was a larger variance of size and shape in non-viable eggs. For all species, larger eggs, or eggs with larger operculum, were more frequently non-viable. However, these differences did not allow for an accurate prediction regarding egg viability.

**Conclusions:**

The strong taxonomic signal present in egg morphology was affected by the level of viability of the eggs. The metric properties as modified in non-viable eggs presented some general trends which could suggest the existence of an optimum phenotype for size and for shape. Globally, viable eggs tended to have intermediate or small sizes, and presented a less globular shape in the Triatomini, or a relatively wider neck in *Rhodnius ecuadoriensis*.

**Electronic supplementary material:**

The online version of this article (10.1186/s13071-018-3104-1) contains supplementary material, which is available to authorized users.

## Background

The species of the subfamily Triatominae (Hemiptera: Reduviidae) are blood-sucking vectors of *Trypanosoma cruzi*, the causative agent of Chagas disease. Currently, more than 150 species have been recognized as potential vectors of the parasite to mammals; however, only a few have significant importance in transmission to humans [1–3]. In Ecuador, 16 species of the Triatominae have been reported, distributed in 20 of the 24 provinces [4, 5]. The infestation index in this country is variable among provinces, ranging between 0.2–29%, with a national average of 2.6% according to the complete analysis of records from the Ministry of Public Health from 2004–2014 [6]. *Rhodnius ecuadoriensi*s (Lent & León, 1958) and *Triatoma dimidiata* (Latreille, 1811) are considered the main vectors in this country. However, other species belonging to the genera *Triatoma* and *Panstrongylus* are increasingly reported as secondary vectors [4].

*Triatoma carrioni* (Larrousse, 1926) is distributed in the southern Andean region of Ecuador (Loja and El Oro) and northern Peru. It occupies a wide range of ecological zones, either arid or humid areas, between 800 and 2242 m above sea level (masl). It is the only species in Ecuador that has been found up to 2242 masl [7]. It may infest human dwellings, primarily in bedrooms as well as peridomestic environments such as chicken nests, guinea pig pens, dog houses, piles of wood, bricks, and firewood [7]. Thus far, the species has not been reported in sylvatic environment (Padilla et al., unpublished data).

*Panstrongylus chinai* (Del Ponte, 1929) is more widely distributed in Ecuador and Peru [4, 8]. In Peru, this species is reported to be the primary household vector in the Department of Piura [9]. In Ecuador, it is reported at altitudes ranging from 175 to 2003 masl, in peridomestic environments (chicken nests, guinea pig pens) as well as in human dwellings (bedrooms) in the southern provinces of Loja and El Oro [4, 7, 10]. Despite considerable sampling efforts, there are no reports of sylvatic populations of *P. chinai* in Ecuador [11].

*Panstrongylus howardi* (Neiva, 1911) is an endemic species restricted to Ecuador in the Manabí Province (central coast region) [12]. It has been associated with rodent nests located between brick piles. Abundant colonies of this species can be found also in wood piles, as well as in the “piñuelas” plant *Aechmea magdalenae*, again in association with nesting places of rodents or of marsupials [13]. Sylvatic specimens have also been reported [14].

Populations of *R. ecuadoriensis* are widely distributed in the southern Andean regions (Loja and el Oro provinces) of Ecuador, and in the central coast, Santo Domingo de los Tsáchilas and Manabí provinces [7, 15]. The species is found also in northern Peru [16]. In Ecuador it occupies domestic, peridomestic and sylvatic habitats [14, 15]. In both countries, abundant sylvatic populations can be found in nests of the Guayaquil squirrel (*Sciurus stramineus*) and the fasciated wren bird (*Campylorhynchus fasciatus*) [5, 14, 17, 18].

The taxonomic classification of the Triatominae was historically based on qualitative morphological descriptions. To introduce more quantitative data and open the field more widely to the study of biological diversity, species in the Triatominae have been studied using quantitative, morphometric techniques. During the last two decades, the geometric approach to morphometrics has become a popular and useful tool in quantifying shape and size variation [19, 20]. This approach has been put into practice in various fields of ecology, evolution, and medicine [21, 22].

Triatomine eggs are oval, elliptical, cylindrical or spherical, slightly asymmetrical forms, and present a smooth convex or ornamented operculum [23, 24]. Egg morphology has been examined mainly on the basis of qualitative characters such as color pattern and structural traits (shape, texture of shell and operculum, exochorial architecture). The quantitative traits of eggs usually were based on traditional morphometric techniques [25–30], while geometric techniques have only recently been introduced [31, 32]. In dwellings, eggs could be found in the cracks of walls, boards of the beds, clothes, chicken nests, and any material accumulated in the domicile and peridomicile [10, 33]. In general, eggs are important to consider because the presence of eggs of some species of the Triatominae in the domicile could be related to its capacity to colonize human dwellings [34], suggesting its importance as a vector. It is therefore relevant to develop egg-based species identification techniques [32].

Many factors could cause artefactual morphometric differences between groups [35]; for the eggs it is important to consider the impact of position, developmental stage, and some other parameters related to the mother and/or the environment [32]. The physiological status of females could influence indeed some traits of their eggs. This possible effect was hereby taken into account by comparing eggs coming from newly-molted females.

The primary justification of our study was to explore the possible interference of egg viability in species distinction. It is a relevant issue because, in some cases, only eggs are collected in the field and some of them could be non-viable eggs. The causes of non-viability are many: eggs could be unfertilized, or even if they are fertilized, they could fail to develop into viable nymph because of unknown genetic or environmental reasons.

As previously shown, geometric techniques of morphometrics applied to viable eggs allow for a very accurate recognition of species or even geographical populations within-species [32, 36]. In four species of Ecuadorian Triatominae, we verified whether the egg-based species morphometric discrimination was affected by the viability of the eggs. We then examined within each species the metric differences between viable (V) and non-viable (NV) eggs.

## Methods

### Insect collection

**Fig. 1:**
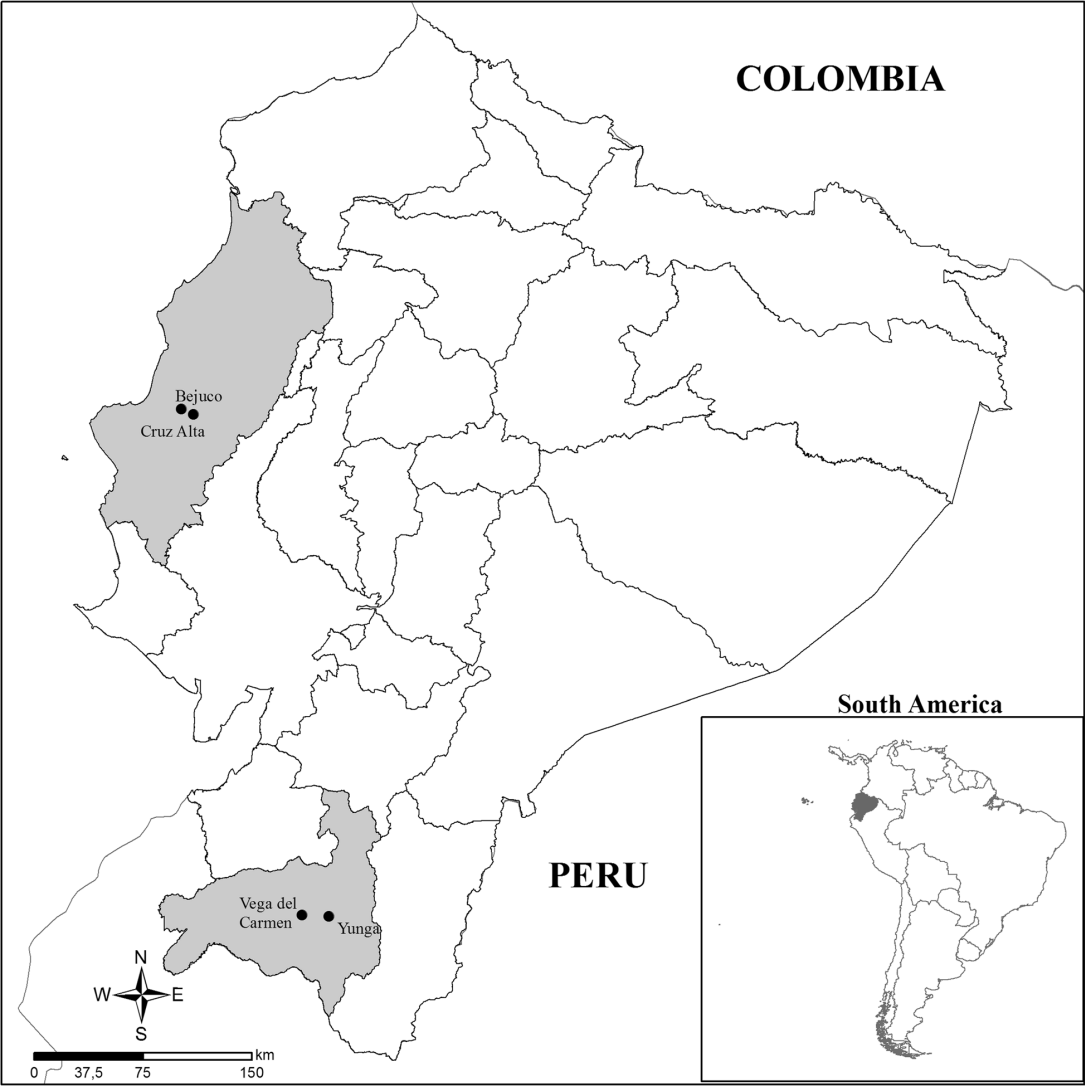
Geographical origin of the specimens in Ecuador. Map showing the location of the communities under study in Loja and Manabí provinces

Specimens were collected in two separate provinces of Ecuador: Loja and Manabí (Fig. 1). In Loja, the houses have roofs of clay tiles, dusty floors, and adobe walls, which provide hiding places and breeding sites for bugs [37]. The houses of Manabí are elevated by wooden stilts and the walls and floors are made of guadúa cane (*Guadua angustifolia*), which allows passage by insects but does not offer hiding places for the bugs [38]. Therefore, in Manabí Province, bugs are usually found in the peridomestic environment.

The peridomestic environment is characterized by chicken and pigeon coops, guinea pig pens, agricultural and household waste, plants used as natural fences, piles of construction materials (stones, wood, bricks), and accumulations of palm fronds (only in Manabí) [15, 38]. In the sylvatic environment in Manabí Province, an abundance of *Phytelephas aequatorialis* is typical, which is cultivated for its nuts used in manufacturing and its leaves for the construction of roofs [38].

The four Ecuadorian species were chosen because of their epidemiological importance for the country. *Rhodnius ecuadoriensis* is one of the most important vectors of Chagas disease in Ecuador, while *T. carrioni*, *P. chinai* and *P. howardi* are considered secondary vectors in their respective provinces [4, 11, 12].

*Panstrongylus chinai* and *T. carrioni* were collected in Loja Province, while *P. howardi* and *R. ecuadoriensis* were collected in the Manabi Province (Table 1). The eggs were obtained from females that had spent various generations (from 4 to 10) in the laboratory. In the case of *T. carrioni*, the eggs were obtained directly from field females collected from domestic habitat. The colonies of *Panstrongylus* spp. and *R. ecuadoriensis* were maintained in the insectary of the Center for Research on Health in Latin America (CISeAL), Pontifical Catholic University of Ecuador (PUCE), under controlled conditions of 25 ± 6 °C, 70 ± 5% relative humidity (RH) for Loja specimens, 27 ± 5 °C, 75 ± 5% RH for Manabí specimens, and a photoperiod of 12:12 h (L:D) for specimens of both provinces. Blood meals were offered every 15 days for 30 min, using immobilized pigeon (*Columba livia*).

**Table 1:** Geographical location and year of collection in communities from which parents were used for egg production

Spp.	Province, community	E	Latitude	Longitude	Altitude	Year	Parents’ origin^a^	Group	*n*
*P. c.*	Loja, Vega del Carmen	P	-4.1137	-79.5928	1086	2009	Laboratory colony (6th generation)	V	34
								NV	22
*T. c.*	Loja, Yunga	I	-4.1225	-79.4284	1622	2015	Field colony (1st generation)	V	21
								NV	12
*P. h.*	Manabí, Bejuco	P	-0.99007	-80.3475	134	2007	Laboratory colony (8th generation)	V	67
								NV	55
*R. e.*	Manabí, Cruz Alta	S	-1.00149	-80.2705	971	2013	Laboratory colony (4th generation)	V	19
								NV	14

*Abbreviations*: *P.c. Panstrongylus chinai*, *P.h. P. howardi*, *T.c. Triatoma carrioni*, *R.e. Rhodnius ecuadoriensis*, *E* ecotopes, *P* peridomicile, *I* intradomicile, *S* sylvatic, *V* viable eggs, *NV* non-viable eggs, *n* number of eggs analyzed by group per species. A total of 244 eggs were analyzed

^a^Provenance of the parents which were used for obtaining the eggs (estimated number of generations according to the life-cycle)

For *P. chinai*, *P. howardi* and *R. ecuadoriensis*, four crosses were conducted and inspected everyday (looking for eggs). Each cross was composed of two females and three males. The eggs of *T. carrioni* came from two field-collected females; they were laid in the collection vials during the transportation of females from the field to the insectary of the center at Quito. Therefore, the eggs came from eight females per species, except for *T. carrioni*. A total of 244 eggs were analyzed: 56 *P. chinai*, 122 *P. howardi*, 33 *T. carrioni* and 33 *R. ecuadoriensis* (Table 1). For each species, the bugs used as genitors came from a single collection (i.e. one house in one locality).

### Egg viability

The eggs were considered as non-viable (NV) if no hatching occurred after 45 days of development. Egg viability (%) was assessed for each species as the ratio of total hatched eggs over total laid eggs. We also estimated the average number of eggs obtained by female per day, and the development time of the four species.

### Morphometric analyses of eggs

As described in Santillán-Guayasamín et al. [32], the eggs were photographed one by one at the same developmental stage and exactly the same position using a MiScope-MIP (www.zarbeco.com) on a platform. The developmental stage was identified by (i) the number of days of embryonic development at photography day and (ii) the presence of visible, darker eye-spots in the anterior operculum zone. The photography day is different between species because of the genus-specific development time. *Panstrongylus* eggs were photographed at the 25 days of development, *Triatoma* eggs at 20–23 days, and *Rhodnius* eggs at 10 days of development [32].

**Fig. 2:**
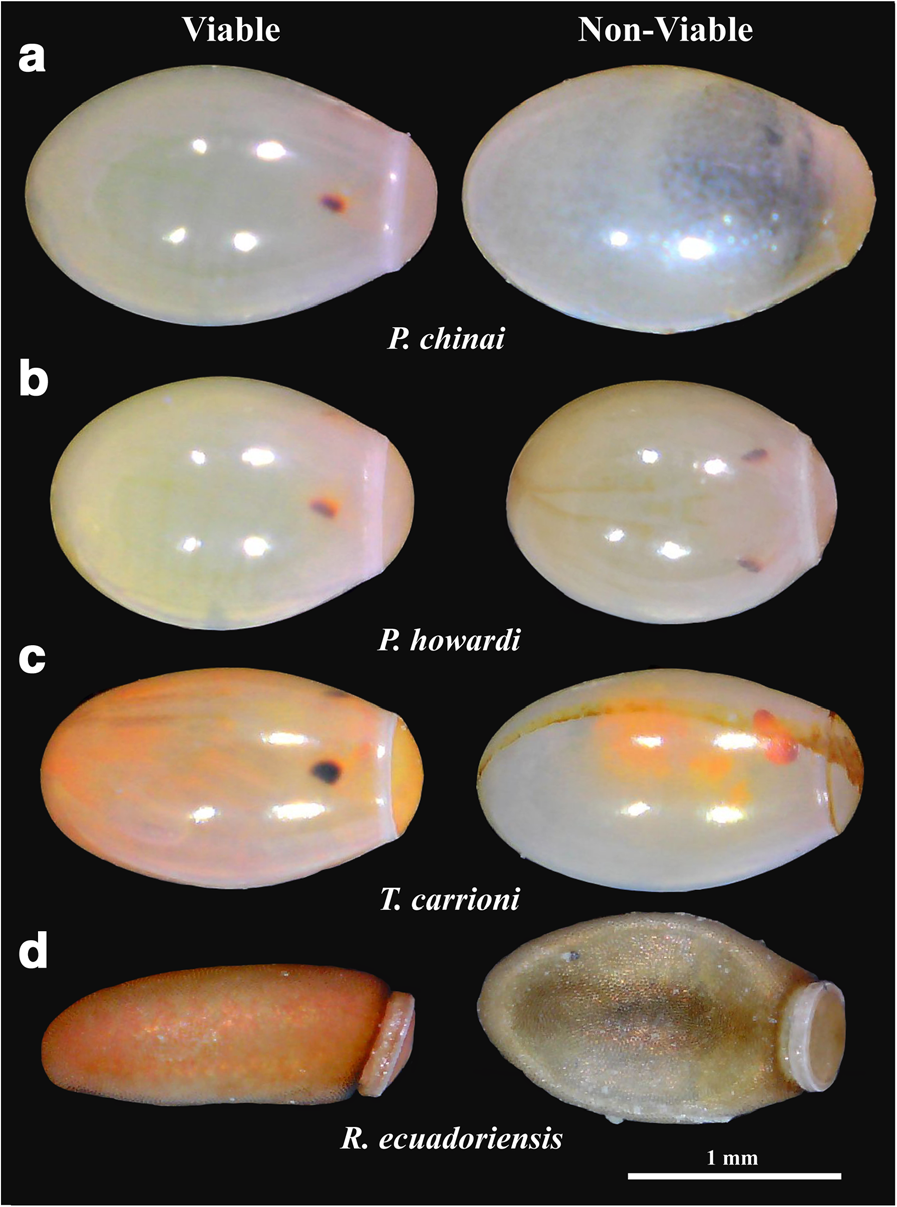
Egg viability. Examples of viable (left) and non-viable (right) eggs of *Panstrongylus chinai*, *P. howardi*, *Triatoma carrioni* and *Rhodnius ecuadoriensis*. The non-viable eggs show eggs with yolk only (lacking of obvious development signals, as in *P. chinai*) (**a**); embryo in normal development (as in *P. howardi*) (**b**); embryo with malformations (development alteration, as in *T. carrioni*) (**c**) or a deflation-like deformation after 25 days of development (as in *R. ecuadoriensis*) (**d**). *Abbreviations*: *P*, *Panstrongylus*; *T*, *Triatoma*; *R*, *Rhodnius*

Eggs of the Triatomini tribe were photographed in ventral position while those of the Rhodniini tribe were photographed in lateral position [32]. The viability status of the eggs was checked after the photographs were taken (days 20–25 for Triatomini, or day 10 for Rhodniini). After this step the NV eggs could be observed developing a progressive deflation-like deformation and/or lacking some obvious development signals (i.e. eye-spots) (Fig. 2).

### Morphometric approaches

We applied two different geometric approaches: the outline-based morphometrics for the complete contour of the egg, and the landmark/semilandmark-based morphometrics for the contour of the operculum (Additional file 1: Table S1) [32]. Both approaches included two main steps: (i) extraction of size and shape variables, which is specific to the technique used, and (ii) discrimination using final shape variables.

#### Egg contour

For shape variable definition, we exclusively used the elliptic Fourier analysis (EFA) [39]. Briefly, the observed contour is decomposed in terms of sine and cosine curves of successive frequencies called harmonics, and each harmonic is described by four coefficients. With this method, the first harmonic ellipse parameters are used to normalize the elliptic Fourier (NEF) coefficients so that they are invariant to size, rotation, and the starting position of the outline trace. By doing this, the three first coefficients become constant (1, 0 and 0) and are not used in the remaining analyses. The fourth coefficient, the one related to the width-on-length ratio of the outline, has been used in our study. The EFA algorithm does not require the points to be equidistant, nor does it require them to be in the same number [40]. The square root of the internal area of the contour (sqrA) and the perimeter of the contour (Per) were computed to estimate the size of the complete egg. For each species, the linear correlation coefficient (r) was computed between these two estimates of size (sqrA and Per).

To accurately represent a closed curve, many harmonics are needed, each one with four coefficients, so that the number of variables would be too numerous relative to the number of individuals. The normalized coefficients (NEF) were thus submitted to a principal component analysis (PCA), and the principal components (PC) or a reduced set of first PC were the final shape variables. This procedure allows for reducing the number of input variables (PC) where necessary.

#### Operculum shape

We used 2 unambiguous landmarks for the Triatomini eggs and 4 unambiguous landmarks for the *Rhodnius* eggs. Between landmarks, we additionally used 8 semilandmarks to capture the external, curved line of the operculum, and 4 to trace the curved boundary between egg and operculum in both types of eggs.

All landmarks and semilandmarks were submitted to partial Procrustes superimposition [41] and semilandmarks then subjected to a sliding procedure [42, 43]. The tangent space orthogonal projections [44] of the aligned configurations were used as input for a principal components analysis (PCA), and the principal components (PC) or a set of first PC were retained as final shape variables. The centroid size (CS) was estimated as the square root of the sum of the squared distances between the center of the landmarks configuration and each individual landmark [45].

### Statistical comparisons

We compared the different species using either the V eggs, NV eggs, or both, and within each species we also compared V and NV eggs. All statistical comparisons considered separately the metric properties (size and shape) of the complete egg and those of the operculum only.

#### Size comparisons

Statistical comparisons of sizes were based on non-parametric, permutation-based tests (1000 cycles) with statistical significance estimated after Bonferroni correction. Using estimates of size, a validated classification procedure was applied based on the maximum likelihood method [36] between either different species or between V eggs and NV eggs.

#### Shape-based discrimination

For the egg contour, each pairwise Mahalanobis distance between groups was computed using the first 32 principal components (PC) of the normalized elliptic Fourier coefficients. For the operculum, Mahalanobis distance was computed using the PC of the tangent space variables. Statistical significance was based on non-parametric, permutation-based tests (1000 cycles), and submitted to the Bonferroni correction. The validated reclassification derived from shape was based on the Mahalanobis distances using PC as input. The latter were used to build an unweighted pair-group method with arithmetic average (UPGMA) tree, using as operating taxonomic units (OTUs) the four species subdivided according to V eggs and NV eggs.

We compared the variance of shape (also called metric disparity, MD) between species as well as between V eggs and NV eggs. It was computed as the trace of the variance-covariance matrix of the final shape variables of each group. Statistical significance of comparisons between MD was based on bootstrap tests (5000 runs) as described by Zelditch et al. [20].

#### Contribution of size to shape-based discrimination

The contribution of size variation to shape variation was estimated through the determination coefficient between the estimator of size and the first shape-derived discriminant factor.

### Software

Collection of landmarks/semilandmarks and pseudolandmarks on eggs, as well as image digitalization and statistical methods (EFA, Procrustes superposition, multivariate analyses) were performed using the CLIC package (http://xyom-clic.eu/). The UPGMA tree was constructed using the *ape* R package (http://ape-package.ird.fr/).

## Results

The primary aim of our study was to evaluate the possible effects of egg viability on the morphometric discrimination between species. The results of this study were divided into: (i) interspecific discriminations, where we checked the influence of NV eggs on species discrimination; and (ii) intraspecific comparison, where we explored possible morphometric differences between viable (V) and non-viable (NV) eggs of the four species.

### Egg viability

The highest developmental time of the eggs was observed in *P. chinai* (30.15 ± 1.13 days), while the lowest time was scored for *R. ecuadoriensis* (18.22 ± 0.94 days). The eggs laid per female varied from 83 (*P. howardi*) to 144 (*P. chinai*) depending on the species. The percentage of V eggs from females reared in the laboratory was lower (from 42 to 79%) than for the field collected eggs (88% for *T. carrioni*) (Table 2).

**Table 2:** For each species: time of development in days, average number of eggs per female per day, number of hatched eggs per female per month, and percentage of viable eggs

Species	Developmental time (days)	Eggs laid/female/day^a^	Eggs hatched	% Viability
*P. c.*	30.15 ± 1.13	4.79 (144)	114	79.17
*P. h.*	28.99 ± 1.38	2.78 (83)	35	42.17
*T. c.*	25.00 ± 0.79	3.77 (113)	99	87.61
*R. e.*	18.22 ± 0.94	3.71 (111)	81	72.97

*Abbreviations*: *P.c. Panstrongylus chinai*, *P.h. P. howardi*, *T.c. Triatoma carrioni*, *R.e*. *Rhodnius ecuadoriensis*

^a^Average number of eggs per female per day, computed from the monthly record of eggs (value in parentheses)

### Interspecific discrimination by size

Since the contour of the egg is smooth, the estimations of size such as the perimeter and the square root of area were correlated with generally high scores (*r* = 92%, *r* = 88% and *r* = 75% for *Panstrongylus* spp., *T carrioni* and *R. ecuadoriensis*, respectively; *P* < 0.001), we thus restricted our size estimator to the square root of the egg area (sqrA).

**Fig. 3:**
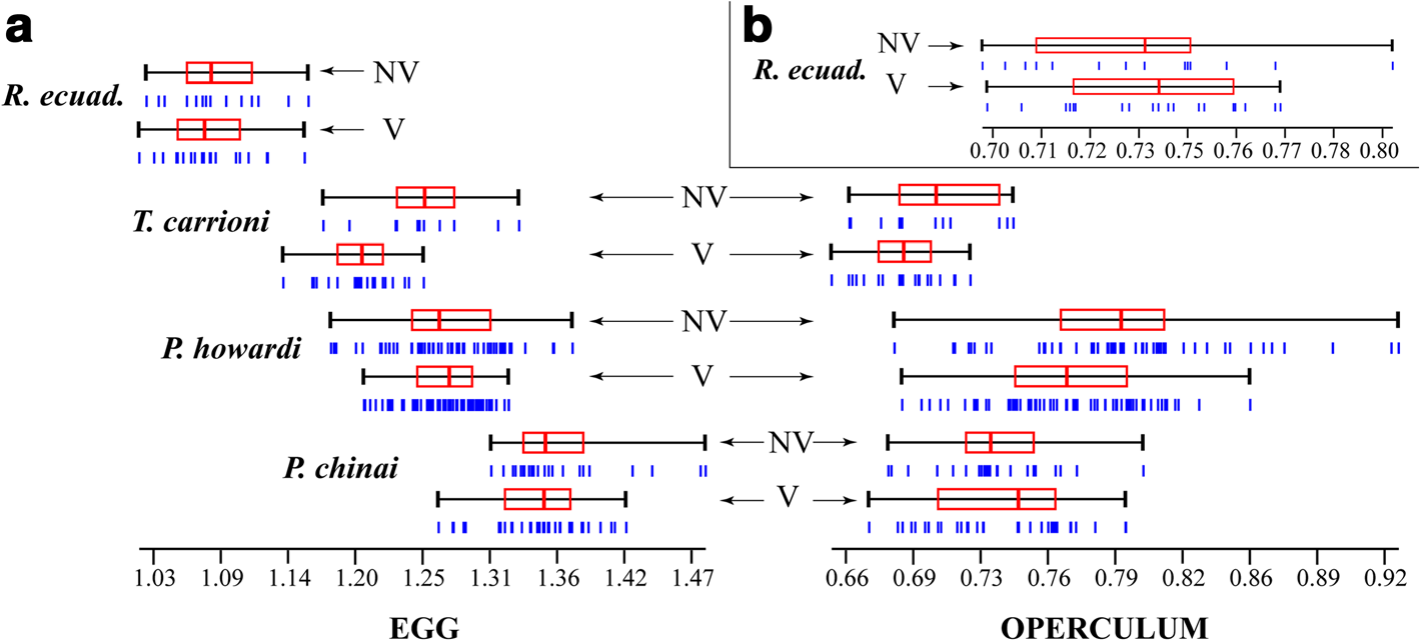
Size variation of egg and operculum. **a** Differences between *Panstrongylus chinai*, *P. howardi*, *Triatoma carrioni* and *Rhodnius ecuadoriensis.* **b** Differences in operculum size between V eggs and NV eggs of *R. ecuadoriensis*. All values are expressed in millimeters. *Abbreviations*: *P*, *Panstrongylus*; *T*, *Triatoma*; *R. ecuad.*, *Rhodnius ecuadoriensis*; V, viable; NV, non-viable

For all comparisons (V, NV and both), *R. ecuadoriensis* was the species with smallest eggs, while *P. chinai* harbored the largest ones. The size of the operculum did not show a strict parallelism with the whole egg, since the largest operculum was observed for *P. howardi*, not for *P. chinai* (Table 3, Fig. 3). Almost all the pairwise comparisons of size between species were found to be significant (*P* < 0.001).

**Table 3:** Mean values of size and standard deviation of the complete egg contour (square root of the area within the egg boundary) and of its operculum (centroid size of the operculum) for viable eggs (V), non-viable eggs (NV) and both

Species		V	NV	*P-*value^*^	V + NV
*P. c.*	Egg	1.34 ± 0.04^a^	1.36 ± 0.05^a^	0.089	1.35 ± 0.04^a^
	Operculum	0.74 ± 0.03^e^	0.74 ± 0.03^d^	0.834	0.74 ± 0.03^d^
*P. h.*	Egg	1.27 ± 0.03^b^	1.27 ± 0.04^b^	0.722	1.26 ± 0.04^b^
	Operculum	0.77 ± 0.03^f^	0.80 ± 0.05^e^	<0.001	0.78 ± 0.04^e^
*T. c.*	Egg	1.21 ± 0.03^c^	1.26 ± 0.04^b^	0.000	1.22 ± 0.04^c^
	Operculum	0.70 ± 0.02^g^	0.71 ± 0.03^f^	0.219	0.70 ± 0.02^f^
*R. e.*	Egg	1.09 ± 0.03^d^	1.09 ± 0.04^c^	0.605	1.09 ± 0.04^d^
	Operculum	0.74 ± 0.02^h^	0.73 ± 0.03	0.678	–

*Abbreviations*: *P. c. Panstrongylus chinai*, *P. h. P. howardi*, *T. c. Triatoma carrioni*, *R. e. Rhodnius ecuadoriensis*

^*^P-values, statistical significance for the comparison of mean sizes between V eggs and NV eggs. If means are different between species, the superscripts (a-h) show different letters. These letters must be read within each column, not between columns. Mean and standard deviation are expressed in millimeters

For the complete egg contour, the variance of size did not show any significant difference between species (*P* ranging between 0.994–0.100). However, for the operculum, in three pairwise comparisons the variance of size showed significant interspecific difference: (i) between *P. chinai* and *P. howardi* for the total sample and for the NV eggs (*P* = 0.004 and *P* = 0.007, respectively); (ii) between V eggs of *P. chinai* and *T. carrioni* (*P* = 0.002); and (iii) between NV eggs of *P. howardi* and *T. carrioni* (*P* = 0.015) (Table 4).

**Table 4:** Variance of size and variance of shape for the egg contour and for the operculum

Species	Viability	Egg, variance		Operculum, variance	
		Size	Shape	Size	Shape
*P. c.*	V	0.0015^a^	0.00017^a,b^	0.0010^a^	0.0016^a^
	NV	0.0022^b^	0.00062^d^	0.0008^c^	0.0040^c^
	P*	0.4360	0.00000	0.427	0.0000
	V + NV	0.0018^c^	0.00035^f^	0.0009^e^	0.0026^e^
*P. h.*	V	0.0008^a^	0.00017^a^	0.0012^a^	0.0025^a^
	NV	0.0019^b^	0.00026^e^	0.0023^d^	0.0024^d^
	P*	0.0010	0.02900	0.046	0.4160
	V + NV	0.0013^c^	0.00021^e^	0.0019^f^	0.0025^f^
*T. c.*	V	0.0008^a^	0.00012^b^	0.0004^b^	0.0010^b^
	NV	0.0019^b^	0.00029^e^	0.0007^c^	0.0028^c,d^
	P*	0.1480	0.00200	0.157	0.0000
	V + NV	0.0017^c^	0.00019^e^	0.0005^e^	0.0022^g^
*R. e.*	V	0.0011^a^	0.00034^c^	0.0004	0.0009
	NV	0.0014^b^	0.00045^d^	0.0008	0.0011
	P*	0.6120	0.03800	0.2370	0.1430
	V + NV	0.0012^c^	0.00037^f^		

*Abbreviations*: *P. c. Panstrongylus chinai*, *P. h. P. howardi*, *T. c. Triatoma carrioni*, *R. e. Rhodnius ecuadoriensis*, *V* viable, *NV* non-viable

*P, statistical significance of the comparison between V eggs and NV eggs for either the variance of size or the variance of shape. Except for *P*, values are variances of either size or shape, for either the egg contour or the operculum. For the egg contour, size was the square root of the area within the egg boundary and shape was the normalized elliptic Fourier coefficients. For the operculum, size was the centroid size and shape was estimated by the tangent space orthogonal projections (see Methods). The variance of shape was computed as the trace of the variance-covariance matrix (see Methods). Different superscripts (a-f) between species indicate significant difference between species. These letters must be read within each column, not between columns. Variance of size is expressed in millimeters

On average, mixing V eggs and NV eggs, species were weakly separated by the size of the egg contour (72%) or by the size of the operculum (65%). Opposite trends between the egg contour and the operculum could be observed when taking into account egg viability to distinguish species (Table 5).

**Table 5:** Percentages of correctly assigned species (ratios between parentheses) for viable eggs (V), non-viable eggs (NV) and both, using either size or shape, egg contour or operculum

Species		Egg			Operculum		
		% V	% NV	% V + NV	% V	% NV	% V + NV
*P. c.*	Si	82 (28/34)	95 (21/22)	87 (49/56)	35 (12/34)	59 (13/22)	45 (25/56)
	Sh	100 (34/34)	73 (16/22)	87 (49/56)	56 (19/34)	64 (14/22)	62 (35/56)
*P. h.*	Si	72 (48/67)	38 (21/55)	61 (74/122)	64 (43/67)	84 (46/55)	71 (87/122)
	Sh	96 (64/67)	93 (51/55)	98 (119/122)	66 (44/67)	80 (44/55)	75 (92/122)
*T. c.*	Si	81 (17/21)	50 (6/12)	70 (23/33)	76 (16/21)	75 (9/12)	76 (25/33)
	Sh	100 (21/21)	100 (12/12)	97 (32/33)	76 (16/21)	42 (5/12)	70 (23/33)
*R. e.*	Si	95 (18/19)	93 (13/14)	91 (30/33)	–	–	–
	Sh	100 (19/19)	100 (14/14)	100 (33/33)	–	–	–
Global	Si	79 (111/141)	59 (61/103)	72 (176/244)	58 (71/122)	76 (68/89)	65 (137/211)
	Sh	98 (138/141)	90 (93/103)	95 (233/244)	65 (79/122)	71 (63/89)	71 (150/211)

*Abbreviations*: *P. c. Panstrongylus chinai*, *P. h. P. howardi*, *T. c. Triatoma carrioni*, *R. e. Rhodnius ecuadoriensis*, *Si* size, *Sh* shape, *Global* total scores across species

### Interspecific discrimination by shape

All pairwise interspecific comparisons of shape (of either eggs or operculum) were highly significant (permutation test, 1000 cycles, *P* < 0.001). For the egg contour, the values of Mahalanobis distances between V eggs were higher than between NV eggs, with an increase going from 42 up to 85%. An opposite trend was observed for the distances derived from the operculum shape. When considering the total sample mixing V eggs and NV eggs, the Mahalanobis distances showed intermediate values.

For all the interspecific comparisons (using V, NV or both kinds of eggs), the reclassification scores obtained from the contour of the whole egg (95%, on average) were better than those obtained from the operculum (71%, on average) (Table 5).

As for size, opposite results between egg contour and operculum shape were observed when distinguishing species using either V eggs or NV eggs. For the egg contour, reclassification scores and Mahalanobis distances were negatively affected when we included the NV eggs in the analysis; for the operculum, however, the reclassification improved when we included NV eggs (Table 5).

**Fig. 4:**
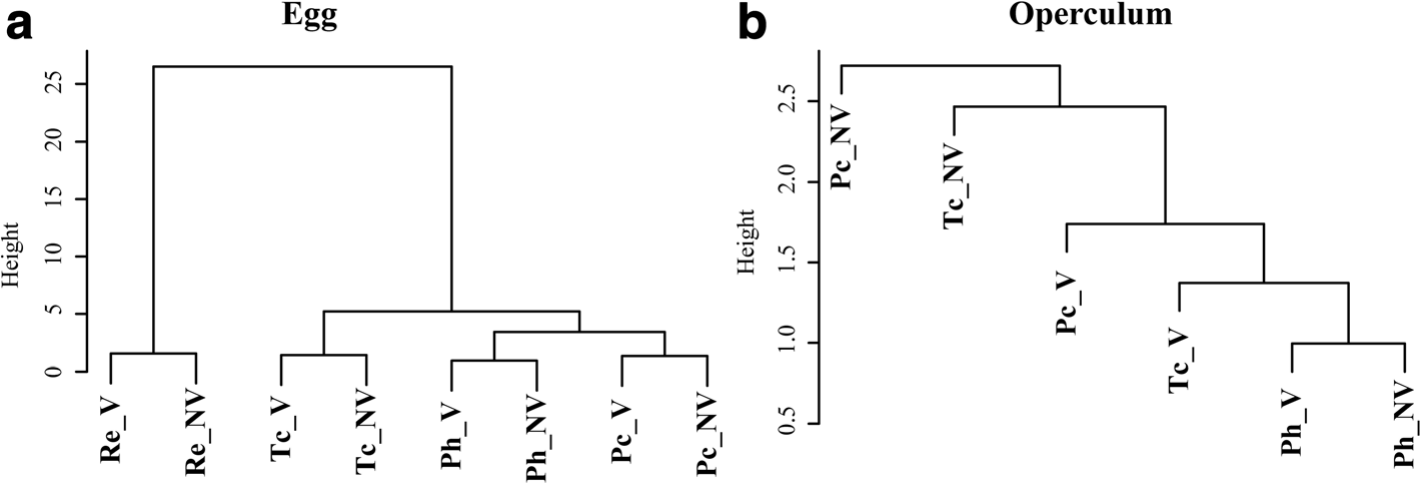
UPGMA phenogram based on shape variation. **a** UPGMA tree inferred from shape of the contour of the complete egg of *Panstrongylus chinai*, *P. howardi*, *Triatoma carrioni* and *Rhodnius ecuadoriensis*. **b** UPGMA tree derived from operculum shape of *P. chinai*, *P. howardi*, and *T. carrioni. Abbreviations*: Re, *Rhodnius ecuadoriensis*; Tc, *Triatoma carrioni*; Ph, *Panstrongylus howardi*; Pc, *P. chinai*; V, viable eggs; NV, non-viable eggs

The UPGMA tree based on the external contour of the eggs showed (i) complete separation between tribes (Rhodniini and Triatomini); (ii) notable difference between genera of the tribe Triatomini (*Panstrongylus* and *Triatoma*); (iii) distinction between species, either for V eggs or NV eggs; and (iv) a constant clustering of V eggs and NV eggs together. On the other hand, the UPGMA tree based on the operculum shape could not produce interspecific relationships in accordance with known phylogenetic relationships when NV eggs were included. For the operculum, the viability status of eggs could affect the representation of interspecific relatedness (Fig. 4).

### Comparisons of size between viable (V) and non-viable (NV) eggs

The sqrA for NV eggs of *P. chinai* or *T. carrioni* showed larger values than observed for V eggs, while no such difference was observed for the eggs of *P. howardi* or *R. ecuadoriensis*. These differences in size between V eggs and NV eggs were generally not statistically significant, except for *T. carrioni* (*P* < 0.001). Considering the operculum only, the size did not significantly vary between groups, except for *P. howardi* where the NV eggs showed larger values (*P* < 0.001) (Table 3). Thus, larger eggs, or eggs with larger operculum, were found more frequently in NV groups, although this trend was not systematically significant.

On the other hand, the variance of egg size showed larger values in NV eggs, although it was significant only for *P. howardi* (*P* = 0.001) (Table 4). Looking at the distribution of sizes (Fig. 3), NV eggs tended to have extreme sizes, either very small or very large, a pattern particularly obvious for *P. howardi*. Similar trends (excepting *P. chinai*, Table 4) which were not significant and less apparent, were observed also for the operculum (Fig. 3).

The validated reclassification of V eggs and NV eggs, based on egg or operculum size, showed relatively low scores on average (between 42–76% and 39–64%, respectively) (Table 6).

**Table 6:** For each species, validated reclassification scores between viable (V) and non-viable eggs (NV)

Species		Egg			Operculum		
		% V	% NV	Global (%)	% V	% NV	Global (%)
*P. c.*	Si	56 (19/34)	45 (10/22)	52	85 (29/34)	32 (7/22)	64
	Sh	67 (23/34)	59 (13/22)	64	72 (24/34)	55 (12/22)	64
*P. h.*	Si	8 (5/67)	95 (52/55)	47	55 (37/67)	73 (40/55)	63
	Sh	70 (47/67)	64 (35/55)	67	60 (40/67)	58 (32/55)	59
*T. c.*	Si	71 (15/21)	83 (10/12)	76	33 (7/21)	50 (6/12)	39
	Sh	71 (15/21)	50 (6/12)	64	86 (18/21)	50 (6/12)	73
*R. e.*	Si	47 (9/19)	36 (5/14)	42	21 (4/19)	71 (10/14)	42
	Sh	42 (8/19)	50 (7/14)	45	68 (13/19)	50 (7/14)	61
Total	Si	34 (48/141)	75 (77/103)	54	55 (77/141)	61 (63/103)	57
	Sh	66 (93/141)	59 (61/103)	62	67 (95/141)	55 (56/103)	63

The reclassification method was based on either size (Si) or shape (Sh), separately. Validated classification, i.e. the percent of correctly assigned individuals (ratio in parentheses)

*Abbreviations*: *Si* size, *Sh* shape, *P. c. Panstrongylus chinai*, *P. h. P. howardi*, *T. c. Triatoma carrioni*, *R. e. Rhodnius ecuadoriensis*, *Global* total scores

### Comparisons of shape between viable (V) and non-viable (NV) eggs

**Fig. 5:**
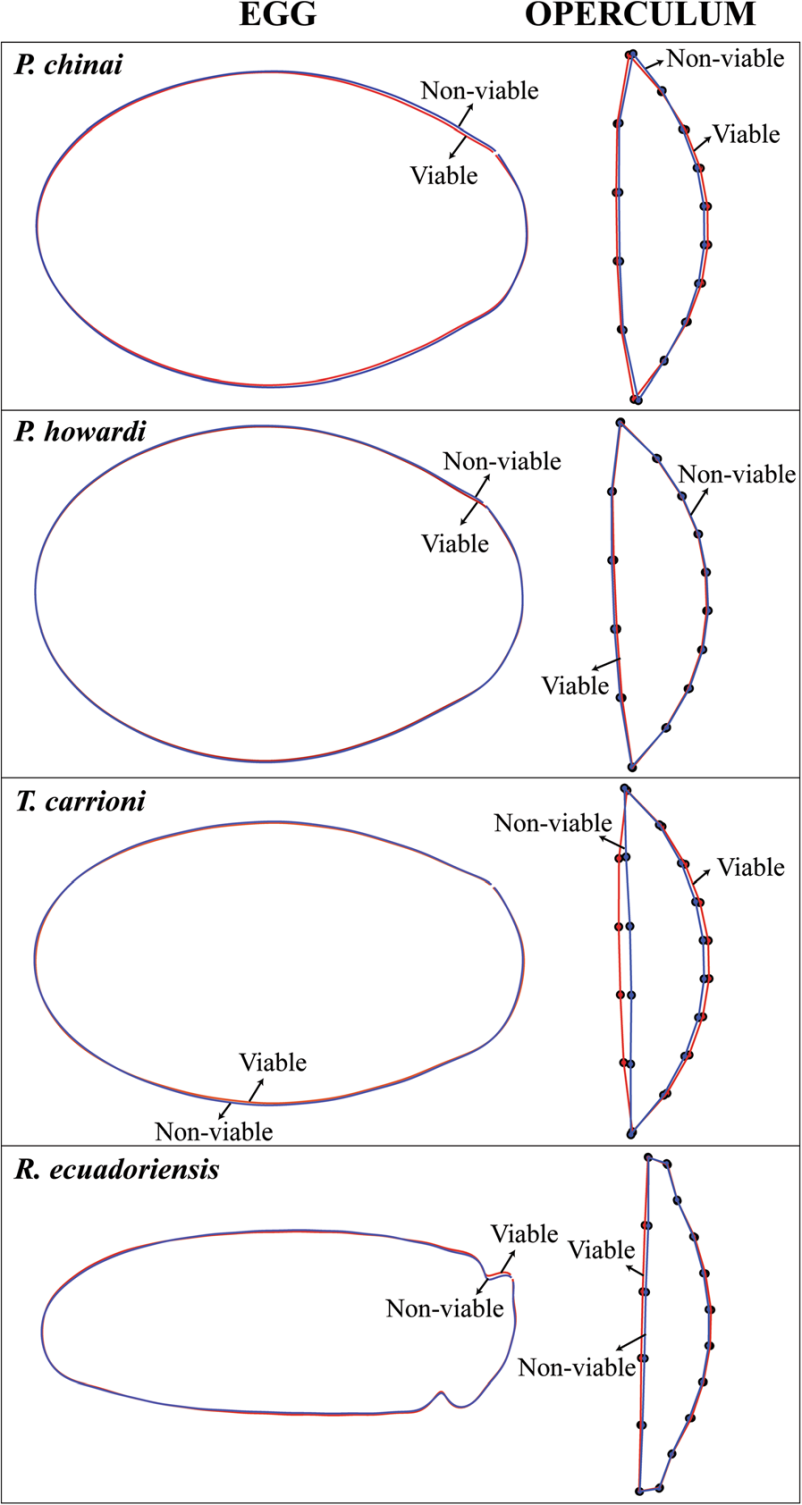
Egg and operculum shape. On the left: egg shape differences between outlines of V eggs and NV eggs of *Panstrongylus chinai*, *P. howardi*, *Triatoma carrioni* and *Rhodnius ecuadoriensis*. In the tribe Triatomini, the shape of V eggs tend to be more slender. On the right: difference in operculum shape between V eggs and NV eggs of *P. chinai*, *P. howardi*, *T. carrioni* and *R. ecuadoriensis*. *Abbreviations*: *P*, *Panstrongylus*; *T*, *Triatoma*; *R*, *Rhodnius*

In Triatomini (“neck-less eggs”), the V eggs tended to be more slender (Fig. 5); this trend was statistically significant only in *P. howardi* (*P* = 0.008). For *R. ecuadoriensis*, the V eggs did not necessarily present a narrower form, but they showed a relatively wider neck than NV eggs (Fig. 5).

The operculum exhibited significant shape difference between V eggs and NV eggs. The shape change was located at the curved limit between the egg and the operculum (Fig. 5). It was statistically significant in *P. chinai*, *P. howardi* and *T. carrioni* (*P* = 0.012, *P* = 0.014 and *P* = 0.013, respectively); however, it was not significant for the Rhodniini (*P* = 0.165).

The reclassification scores between V eggs and NV eggs based on the contour of the whole eggs were lower (between 45–67%) than the ones based on the operculum (between 59–73%) (Table 6). In all species, the metric disparity (MD, variance of shape) of the NV egg contour was significantly larger (1.5 to 3.7 times larger) than the one of V eggs (*P* ranging between 0.038– < 0.001). For the operculum, the MD of NV eggs was larger, although statistically significant only in *P. chinai* and *T. carrioni* (*P* < 0.001 for both species) (Table 4).

Except for the operculum of *P. howardi* (20%), the influence of size variation on shape distinction was low, both for the egg and the operculum (from 0.3 to 6.9%).

## Discussion

This study is motivated by the need for a more precise taxonomic identification of some potential vectors whatever the stage of development available, either adult, nymph or egg. We focused here on the taxonomic interest of eggs according to their viability. The use of eggs is relevant because, in some cases, only eggs (and nymphs) are found in human dwellings [46], and eggs were recently shown to be highly discriminant between species and populations [32, 36].

The diversification of egg morphology among species of the Triatominae is likely to be primarily under genetic determinism. However, various environmental and artefactual causes could interfere with species divergence. Among them, in addition to the developmental stages and the position of the egg [32], we could cite the sampling conditions (time and place of collection), the mother age, the source of blood of the genitors and their feeding frequency, and the origin of the colonies (parents from laboratory colonies or from the field) [47–52]. These factors primarily affect the size of organisms; for this reason the morphometric distinction between taxa was based on shape variation, not on size variation, even if shape distinction was not completely free of some residual size influence (allometric residue). Moreover, these confusing factors were tentatively ruled out here by selecting controlled laboratory conditions (except for *T. carrioni*) and, for each species, by selecting new-molted genitors coming not only from the same locality, but also from the same house.

In these conditions, we could focus better on another possible interference: egg viability. At the time of development as selected by Santillán-Guayasamín et al. [32] to capture egg morphometric data in the best conditions, it is not possible to predict the future of each egg: viable (V) and non-viable (NV) eggs are mixed. Our study tries to estimate the effect of egg viability on its metric properties. Even with a relatively low number of female genitors (eight per species), it was possible to detect some trends.

We observed the highest percentage of viability for the eggs laid by field females (see *T. carrioni*, Table 2). The eggs of females that spent a few generations in the laboratory showed lower viability scores, especially for the females transported from a very different natural environment (see *P. howardi*, Table 2).

The hatching success scores used in this study provided only a partial indication of fitness. However, our study was not designed to understand the causes of egg viability, rather its protocol was set up to describe, and possibly to understand, any association with modified metric properties. Our main question was: could the viability status of the egg affect its morphology to such an extent that species distinction was compromised? We also checked whether there was any typical difference in the size and in the shape of V eggs and NV eggs.

Using V eggs only, the shape of the complete contour could recognize almost perfectly (98%) the four species, while the interspecific comparisons limited to NV eggs or mixing both kinds of eggs reached a consistently lower score (90 and 95%, respectively). For the operculum, the shape was not highly discriminant between species. Unexpectedly, the inclusion of NV eggs in the discrimination increased the classification scores between species.

Thus, in spite of variation according to species, our data suggest that the viability of eggs could modify the interspecific discrimination. Although there were differences in egg shape and size between V eggs and NV eggs, it was not possible to accurately recognize them. When based on shape, the operculum or the complete egg contour produced similar but low reclassification scores between V and NV eggs (62 and 63%, respectively; see Table 6). When based on size, the reclassification scores were lower than those based on shape (54 and 57%, respectively, on average).

The difference in size between V eggs and NV eggs could be related to the consumption of yolk during the development [53, 54], or to an insufficient amount of yolk provided by the mother [49]. In that case, size modification would be the consequence of pathological changes in the egg development. However, physical properties of eggs may also influence their viability by altering resistance to tensile stresses and thus to breakage [55], or by modifying gas exchange (O_2_, CO_2_ and water vapor) between the environment and the developing embryo [56].

The variation in egg size probably reflects adaptive processes for each species in response to environmental heterogeneity [57, 58]. In this regard, our observations could suggest that there is an optimal phenotype for the size. Indeed, the distribution of operculum and egg size between V eggs and NV eggs supported the idea that the eggs of *P. chinai*, *T. carrioni* and *R. ecuadoriensis* could be subject to directional selection in which smaller individuals had a higher probability of surviving than larger ones. However, the eggs of *P. howardi* were apparently subject to stabilizing selection, where either large or small eggs suffered a higher risk of death than eggs of intermediate size [59, 60]. If confirmed, these features would represent another distinction between the very close species *P. chinai* and *P. howardi* (Villacís et al., in prep.).

## Conclusions

To distinguish between species, size did not prove to be a good discriminant character for either the operculum or complete egg. Regarding shape, using either V eggs or NV eggs, the operculum did not appear to be a reliable source for interspecific recognition (65 and 71% of correct assignments, respectively). The complete contour of the egg provided much more satisfactory species classification (95% of correct assignments, on average), with better scores comparing V (98%) than NV (90%) eggs. Thus, species recognition by the complete contour of the egg was very high. We showed that it was affected by the viability status of the egg. A constant metric change from V to NV eggs observed for all species was a larger variance of size and shape in the NV group. For all species, larger eggs, or eggs with larger operculum, were found more frequently in the NV group, suggesting that an optimum phenotype could be found preferably among intermediate or small sizes, presenting a less globular shape in the Triatomini, or a relatively wider neck in *R. ecuadoriensis*. However, it was not possible to use the metric properties to perform an accurate prediction about egg viability.

## Additional file

Additional file 1: **Table S1.** Database. Coordinates of the complete contour of the eggs (outline-based method) and of the operculum (landmark/semilandmark-based approach). (XLSX 558 kb)

## Abbreviations

CS: Centroid size
EFA: Elliptic Fourier analysis
MD: Metric disparity
NEF: Normalized elliptic Fourier
NV: Non-viable
OTUs: Operating taxonomic units
PC: Principal component
PCA: Principal components analysis
sqrA: Square root of the internal area of the contour
UPGMA: Unweighted pair-group method with arithmetic average
V: Viable
